# Editor’s Choice Articles in the Electronic Materials Section of *Materials* in 2025

**DOI:** 10.3390/ma19091825

**Published:** 2026-04-29

**Authors:** Fabrizio Roccaforte

**Affiliations:** Consiglio Nazionale delle Ricerche—Istituto per la Microelettronica e Microsistemi (CNR-IMM), Strada VIII, n.5—Zona Industriale, 95121 Catania, Italy; fabrizio.roccaforte@imm.cnr.it

The “Electronic Materials” Section of *Materials* is dedicated to publishing high-quality contributions advancing both our fundamental understanding and practical implementation of materials for electronic applications. In 2025, seven papers published in the Section [1,2,3,4,5,6,7] were selected as Editor’s Choice Articles in recognition of their technical quality, scientific visibility, and relevance to their respective field. These contributions address critical challenges in materials synthesis, design, interfacial control, device engineering, and reliability. Collectively, they provide a representative perspective on the evolving directions of current electronic materials research. More broadly, they illustrate how this field is increasingly being shaped by the integration of fundamental materials science with technological applications.

One of the most relevant aspects across the selected papers is the central role of interface engineering in determining device performance. Over the last few years, interface preparation has become a decisive issue in emerging semiconductor technologies beyond silicon. As an example, in wide-bandgap (WBG) semiconductors, such as silicon carbide (SiC) [8] and gallium nitride (GaN) [9], which are progressively replacing silicon in many power electronic applications, the electrical behavior of relevant interfaces (e.g., Schottky and MOS junctions) is strongly governed by the surface preparation, interfacial chemistry, defect density, and dielectric quality [10,11,12]. In recent years, it has become increasingly clear that the optimization of dielectric/semiconductor interfaces is one of the principal routes for improving channel mobility, threshold-voltage stability, and long-term reliability in next-generation WBG power devices [10,11,12].

Besides conventional bulk semiconductors, two-dimensional materials, such as graphene and transition metal dichalcogenides (TMDs), have been the subject of intensive investigation over the last two decades [13]. They can also form heterostructures and layered materials composed of crystalline sheets bonded by van der Waals (vdW) interactions. Among the possible applications of these systems, recently, the integration of graphene and MoS_2_ with conventional semiconductors (such as Si, Ge, SiC and GaN) has been proposed, with the aim of enhancing the performance of both electronic/optoelectronic devices and sensors [14]. The electronic properties of these layered semiconductors can be tailored via nanoscale contact engineering, dielectric integration, and van der Waals interface control. In this context, in the field of quantum heterostructures, *Materials* also highlighted in 2025 the work by Kirti et al. [1], who demonstrated the optimization of in situ superconducting Al/InAs hybrid heterostructures on GaAs, establishing an important materials platform for scalable quantum electronic circuits. Their study highlights how precise control of superconductor–semiconductor interfaces can directly influence coherence, reproducibility, and device integration. A related perspective was presented by Hong [5], who reported that graphene–MoS_2_ lateral contacts can significantly reduce contact resistance while enhancing charge transport in two-dimensional electronic systems. This finding reinforces the importance of contact design as a limiting factor in the practical implementation of atomically thin materials.

Another major theme is continued progress in advanced optoelectronic and memory materials. High-impact regular and review papers continuously highlight how defect engineering, dimensionality control, and hybrid integration are redefining the performance limits of photodetectors, neuromorphic memories, and multifunctional electronic platforms [15,16]. Jiang et al. [3] demonstrated an all-carbon diamond solar-blind detector with graphene electrodes, revealing the potential of wide-bandgap materials for operation in demanding environments requiring both high sensitivity and chemical stability. In parallel, Hwang et al. [2] showed how plasma-assisted atomic layer deposition (ALD) can be used to tailor HfO_2_-based dielectric and TiO_2_ channel layers, leading to improved charge-trapping memory performance. Together, these studies illustrate how precise control of materials processing can directly translate into enhanced device functionality.

From a technological perspective, plasma-enhanced atomic layer deposition (PEALD) has emerged as a particularly versatile approach to the growth of conformal ultrathin dielectrics and interfacial layers [17]. Recent studies have demonstrated that even in the early growth stages of Al_2_O_3_ deposition on AlGaN/GaN heterostructures, PEALD can strongly influence nucleation behavior and electrical uniformity at the dielectric/semiconductor interface [18]. Complementary work on more complex gate stacks (e.g., Al_2_O_3_/HfO_2_ nanolaminated) on SiC further showed the potential of PEALD for engineering dielectric performance in WBG devices [19]. Such developments are closely connected to current advances in GaN technology, where dielectric integration by tuning the deposition mode and sequence [20] and the introduction of appropriate interfacial layers [21] have become strategies for passivating native defects and improving interface stability.

Among the 2025 Editor’s Choice Articles, the review by Abramkin et al. [4] on electrochemical silicon deposition offers a valuable assessment of electrolyte systems suitable for industrial implementation. This work is particularly timely, because sustainable and lower-temperature processing routes are becoming increasingly important in semiconductor manufacturing. Beyond conventional microelectronics, electrochemical routes are increasingly being explored as enabling technologies for low-energy semiconductor processing and for the selective growth of silicon in architectures that are difficult to achieve via conventional vapor-phase methods.

Finally, energy efficiency in power electronics conversion is a strategic field (automotives, renewable energies, AI data-centers, etc.), where progress in materials science can contribute to a reduction in overall global energy consumption. Lisik and Podgórski [6,7] examine the advantages and limitations of the superjunction MOSFET concept through a rigorous combination of analytical theory and numerical simulation. Their two-part contribution deepens the understanding of the physical constraints that govern next-generation high-voltage power devices and illustrates the continued relevance of fundamental device physics in materials-orientated research. Similar design concepts are now being widely explored in SiC power devices, where researchers are seeking to overcome the traditional limits imposed by the Baliga figure of merit through superjunction-like architectures, channel engineering, and ion-implantation strategies [22,23]. These developments indicate that the historical evolution previously observed in silicon electronics [24] is now extending into WBG semiconductor technology.

The diversity of these Editor’s Choice Articles reflects the inherently interdisciplinary nature of electronic materials research, and of the “Electronic Materials” Section of the journal. Spanning quantum heterostructures, two-dimensional materials, optoelectronic systems, memory technologies, and power electronics, the selected papers collectively demonstrate how advances in materials science continue to drive innovation at the device level. At the same time, they show that progress in this field is increasingly defined not only by the discovery of new materials but also by the ability to understand interfaces, control defects, and develop scalable fabrication strategies.

Looking ahead, progress in this field will increasingly depend on the integration of different materials with scalable processing methods, predictive modeling, and system-level optimization. The contributions highlighted in 2025 exemplify this direction and underscore the scientific standards that the “Electronic Materials” Section of *Materials* seeks to promote. Beyond recognizing individual excellence, these Editor’s Choice Articles also provide a broader snapshot of research directions that are likely to shape the next generation of electronic materials research.
